# Synaptic Long-Term Potentiation and Depression in the Rat Medial Vestibular Nuclei Depend on Neural Activation of Estrogenic and Androgenic Signals

**DOI:** 10.1371/journal.pone.0080792

**Published:** 2013-11-12

**Authors:** Mariangela Scarduzio, Roberto Panichi, Vito Enrico Pettorossi, Silvarosa Grassi

**Affiliations:** Dipartimento di Medicina Interna, Sezione di Fisiologia Umana, Facoltà di Medicina e Chirurgia, Università di Perugia, Perugia, Italy; Max-Delbrück Center for Molecular Medicine (MDC), Germany

## Abstract

Estrogenic and androgenic steroids can be synthesised in the brain and rapidly modulate synaptic transmission and plasticity through direct interaction with membrane receptors for estrogens (ERs) and androgens (ARs). We used whole cell patch clamp recordings in brainstem slices of male rats to explore the influence of ER and AR activation and local synthesis of 17β-estradiol (E2) and 5α-dihydrotestosterone (DHT) on the long-term synaptic changes induced in the neurons of the medial vestibular nucleus (MVN). Long-term depression (LTD) and long-term potentiation (LTP) caused by different patterns of high frequency stimulation (HFS) of the primary vestibular afferents were assayed under the blockade of ARs and ERs or in the presence of inhibitors for enzymes synthesizing DHT (5α-reductase) and E2 (P450-aromatase) from testosterone (T). We found that LTD is mediated by interaction of locally produced androgens with ARs and LTP by interaction of locally synthesized E2 with ERs. In fact, the AR block with flutamide prevented LTD while did not affect LTP, and the blockade of ERs with ICI 182,780 abolished LTP without influencing LTD. Moreover, the block of P450-aromatase with letrozole not only prevented the LTP induction, but inverted LTP into LTD. This LTD is likely due to the local activation of androgens, since it was abolished under blockade of ARs. Conversely, LTD was still induced in the presence of finasteride the inhibitor of 5α-reductase demonstrating that T is able to activate ARs and induce LTD even when DHT is not synthesized. This study demonstrates a key and opposite role of sex neurosteroids in the long-term synaptic changes of the MVN with a specific role of T-DHT for LTD and of E2 for LTP. Moreover, it suggests that different stimulation patterns can lead to LTD or LTP by specifically activating the enzymes involved in the synthesis of androgenic or estrogenic neurosteroids.

## Introduction

Growing evidences show that synaptic plasticity may be rapidly modulated in different areas of the brain by sex steroids like 17β-estradiol (E2), testosterone (T) and 5α-dihydrotestosterone (DHT), either produced in the gonads or locally synthesised in the brain [1-6]. Synthesis of sex neurosteroids depends on neural conversion of cholesterol into T that is then transformed into E2 and DHT by action of P450-aromatase and 5α-reductase enzymes, respectively [6-15]. The rapid action of these neurosteroids involves a direct interaction with specific membrane receptors for estrogens (ERs: ERα and ERβ) and androgens (ARs) [16-22].

Our previous studies in slice demonstrate opposite long-term synaptic effects in the neurons of the medial vestibular nucleus (MVN) by exogenous administration of E2 or DHT [23,24]. In particular, E2 induced long-term potentiation (LTP) of synaptic response to vestibular afferent stimulation while DHT caused long-term depression (LTD). Interestingly, these effects were also produced by administration of T depending on its transformation into estrogenic or androgenic metabolites [23]. We have recently found by whole-cell patch recordings that the opposite effects of E2 and DHT can occur in the same MVN neuron, a result that is supported by the immunohistochemical evidence of co-localization of ERs and ARs in the MVN neurons [25]. Since it has been shown that the sex neurosterogenesis may be driven by synaptic stimulation involving Ca^2+^ influx through N-methyl-D aspartate receptor (NMDAR) [11-14,26,27], our main purpose was to understand whether and how synthesis of estrogenic and androgenic neurosteroids can be associated with activity dependent induction of NMDAR mediated LTP and LTD in the MVN. We know by our previous field potential recordings that the local synthesis of E2 is implied in LTP induced by afferent high frequency stimulation (HFS) in the MVN and hippocampus CA1 area [28,29]. Now the question is whether different patterns of stimulation may induce opposite long-term effects through activation of androgen synthesising pathway. This is suggested by our recent observation in hippocampus slices showing that induction of LTD by low frequency stimulation depends on activation of the ARs (unpublished data). Therefore, we hypothesised that in the vestibular neurons the direction of the long-term effects in response to different patterns of afferent stimulation is due to the characteristic of the stimulus that may specifically activate the estrogenic or androgenic pathway by facilitating the conversion of T into E2 for LTP, or into DHT for LTD. To demonstrate this hypothesis, we designed this whole cell patch study in which, according to our recent finding [30], we induced LTP or LTD in the MVN neurons by varying the pattern (inter-burst interval and burst number) of high frequency burst stimulation, and we assessed the influence of androgenic and/or estrogenic neurosteroids on these opposite long-term effects by using either the antagonists of ARs and ERs or inhibitors of the DHT and E2 synthesising enzymes. Since MVN synaptic changes like LTP and LTD play a role in visuo-vestibular calibration and vestibular compensation [31,32] in response to different afferent signals, it would be interesting to find out the role of different sex neurosteroids in determining the sign of long-term changes and gain insight into their possible importance in adaptive vestibular overloading and pathological condition.

## Materials and Methods

### Ethics statement

All procedures on animals were performed in strict accordance with protocols approved by the Ethical Committees of the University of Perugia, in compliance with the guidelines of the Italian Ministry of Health, national laws on animal research (Legislative Decree 116/92) and The European Communities Council directive on animal research (N. 86/609/EEC). All efforts were made to minimize the number of animals used and their suffering. 

### Slice preparation and whole cell patch clamp recordings

The study was conducted in brainstem slices prepared from male Wistar rats (16-26 days old, Harlan, Italy). We used male rats to avoid the influence of cyclic estrogenic fluctuation on the induction of synaptic plasticity in the MVN neurons, given that previously we demonstrated that HFS can induce LTP or LTD in female rat, depending on the phase of estrous cycle [33,34]. Following anaesthesia with Avertin (i.p. 250 mg/Kg), the animals were decapitated and brainstem rapidly removed into ice-cold modified high sucrose artificial cerebrospinal fluid (ACSF) of composition (mM): KCl, 2.5; NaH_2_PO_4_, 1; MgSO_4_, 2; CaCl_2_, 0.5; D-glucose, 11; NaHCO_3_, 26.2 and sucrose 238, saturated with 95% O_2_ and 5% CO_2_. Transverse slices (300 µm), containing the MVN, were cut using a vibratome (Series 1000 plus starter CE, Vibratome, St. Louis, MO, USA) and were incubated for at least 1h before recording in warmed (30 ± 1°C) ACSF containing (mM): NaCl, 124; KCl, 3; KH_2_PO_4_, 1.25; NaHCO_3_, 26; CaCl_2_, 2.1; MgSO_4_, 1.7; D-glucose 10 and L-ascorbate 2, saturated with 95% O_2_ and 5% CO_2_, (pH ~7.4). The submerged recording chamber was perfused with warmed (30 ± 1°C) and oxygenated ACSF at a rate of 2 ml/min. 

**Figure 1:**
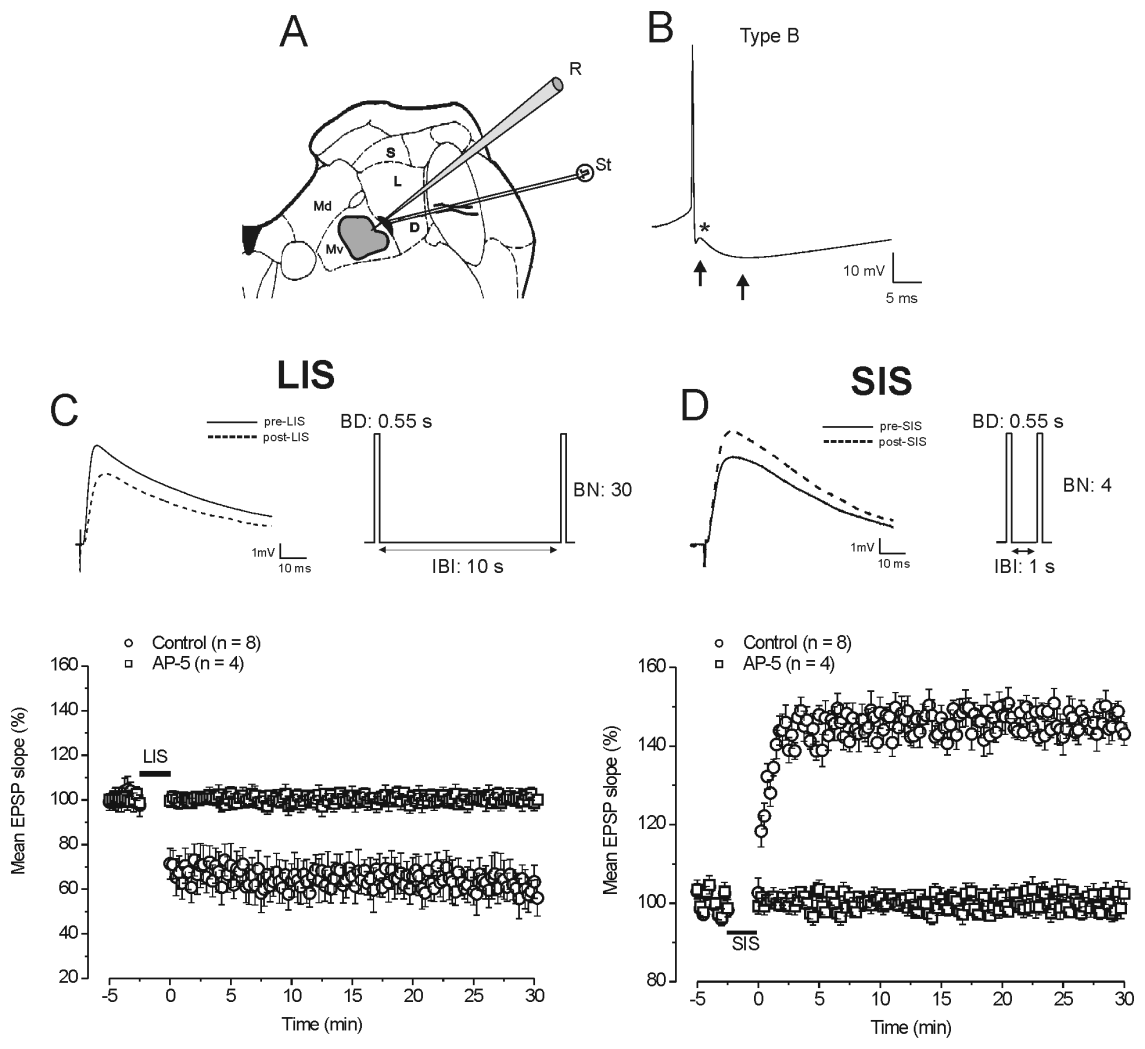
Long-term potentiation and depression are induced by different stimulation patterns at the vestibular afferent synapses of the MVN type B neurons. **A**. Schematic drawing of rat brainstem slice showing position of stimulating electrode (black area) and the recording loci (grey area) in the ventral portion of the MVN. Abbreviations: D, descending vestibular nucleus; Md, dorsal part of the MVN; Mv, ventral part of the MVN; L, lateral vestibular nucleus; S, superior vestibular nucleus; R, recording and St, stimulating electrodes. **B**. Representative action potential recorded from type B neuron displaying an early fast and a late slow AHP (arrows), and the ADP (asterisk). **C**. LTD of EPSP induced by long-interval stimulation (LIS). (Top-left) Averaged traces (n = 20) of EPSPs evoked in type B neurons before (thin trace: pre-LIS) and after LIS (dashed traces: post-LIS). (Top-right) Schematic drawing of LIS protocol with burst duration (BD) = 0.55 s, burst number (BN) = 30 and inter-burst interval (IBI) = 10 s. (Bottom) Time courses of the LIS effects on the slope of EPSP in control condition (circles) and in the presence of AP-5 (squares). In this and following figures each point represents the mean ± SE (from the number of neurons reported in the graph legend) of EPSP slope measured every 15 s and expressed as percentage of EPSP baseline values. **D**. LTP of EPSP induced by SIS. (Top-left) Averaged traces (n = 20) of EPSPs evoked before (thin trace: pre-SIS) and after SIS (dashed traces: post-SIS). (Top-right) Schematic drawing of SIS protocol with BD = 0.55 s, BN = 4 and IBI = 1 s. (Bottom) Time courses of the SIS effects on the EPSP slope in control condition (circles) and in the presence of AP-5 (squares). In this and the subsequent figures the bars indicate stimulation delivery time, so that the negative values represent the times before the start of stimulation and the positive ones those after the end of stimulation.

For each animal we used 2-3 slices prepared from the middle portion of the MVN (about 1.6 mm of the rostro-caudal nucleus length), corresponding to the vestibular nerve root. Neurons from the ventral part of the MVN were visualized by means of a 60x water immersion objective mounted on an upright microscope (Eclipse FN 1, Nikon, Tokyo, Japan), fitted with an analogue video camera (WAT-902B, Watec, Japan). They generally had ovoid soma (~15 μm in diameter) and at least two processes visible. Whole-cell patch recordings were obtained using thick-walled borosilicate glass pipettes (Harvard Apparatus, Holliston, MA, USA) pulled on a P-97 puller (Sutter Instruments, Novato, CA, USA) with a tip resistance of 6-10 MΏ in ACSF when filled with intracellular solution containing (mM): K-gluconate, 145; MgCl_2_6H_2_O, 2; HEPES, 5; EGTA, 0.1 and K_2_ATP, 5 (pH 7.2-7.3; osmolarity adjusted to ~290 mOsm). Whole cell patch clamp recordings were performed using an Axoclamp-2B amplifier (Axon Instruments, Foster City, CA, USA), filtered at 3 kHz and digitized at 10 kHz with an Axon Digidata 1440 A interface controlled by Clampex 10.2 software (Axon Instruments, Foster City, CA, USA). All neurons included here were type B neurons recognized by the shape of their action potential (AP) and in particular based on the presence of a dual component afterhyperpolarization (AHP) with an afterdepolarization potential (ADP) (Figure 1B) [35-37]. We chose to analyse only type B neurons since we found that glutamatergic synaptic response in type-A neurons are not influenced by E2 [24].

Excitatory postsynaptic potential (EPSP) was evoked by stimulating the primary vestibular afferents with a bipolar home-made Pt/Ir-stimulating electrode placed in a narrow zone at the medial border of the lateral or descending vestibular nucleus (Figure 1A), which is the point where a bundle of vestibular fibres enter the MVN. The EPSP was recorded in current clamp mode with the membrane potential (Vm) held at -75 mV by negative holding current to suppress spontaneous neuron discharge. All the recordings were performed under continuous perfusion of ACSF containing picrotoxin (100 μM) and strychnine (1 μM) (Sigma-Aldrich, St Louis, MO) to block spontaneous GABA_A_ and glycine mediated inhibitory post-synaptic currents.

### Stimulus parameters

Test stimulation consisted of bipolar voltage pulses (intensity 5-25 V, duration 70 μs) delivered at a frequency of 0.06 Hz using the stimulus isolation unit ISO-Stim 01D (NPI, Germany) driven by the computer. The stimulus intensity was chosen so that the amplitude of evoked EPSP was 40-60% of the maximum at both stimulus polarities, as determined by an input-output curve. Accordingly with previous studies [30,38] we induced LTD or LTP by using different patterns of burst (0.55 s duration) stimulation at 100 Hz. LTD was induced by 30 bursts repeated with 10 s inter-burst interval (long-interval stimulation, LIS, Figure 1C) and LTP by 4 bursts repeated with 1 s inter-burst interval (short-interval stimulation SIS, Figure 1D).

The type B neurons tested were allowed to fire normally during the application of the induction protocol (current clamp mode, resting Vm = -50 mV). In each single slice, only one neuron was analysed.

### Drugs

Drug used included the antagonists for ARs (flutamide, 100 nM), ERs (ICI 182,780, 100 nM), and NMDAR (D-(-)-2-amino-5-phosphonopentanoic acid (AP-5, 50 μM) and the inhibitors of the enzymes 5α-reductase (finasteride, 1 μM) and P450-aromatase (letrozole, 100 nM). All the drugs were purchased from Sigma-Aldrich (St Louis, MO). Flutamide is commonly used to block ARs but it can also influence GABAergic transmission, because of its similarity with benzodiazepines [39]. However, we can exclude this possibility since the concentration used was much lower than that reported to have anti-convulsant effects [39] and all the recordings were performed under picrotoxin (100 μM) to block spontaneous GABA_A_ mediated inhibitory post-synaptic currents. Concerning ICI 182,780 it is a well known antagonist of nuclear ERs, however it also acts as a membrane ER antagonist mediating rapid estrogenic effects [40,41]. Thus, we used ICI to block ERα and ERβ localized at the cell membrane [22].

Stock solutions of flutamide (10 mM), letrozole (10 mM), ICI 182,780 (1 mM) and finasteride (100 mM) were dissolved in dimethylsulphoxide (DMSO) while stock solution of AP-5 (10 mM) was dissolved in distilled water. The drugs were applied by dissolving them to working concentrations in oxygenated ACSF (containing picrotoxin and strychnine) and were perfused at a rate of 2ml/min. Total replacement of the medium in the chamber occurred within 1 min. In all the experiments using these different drugs the baseline analysed 10 min after the beginning of drug application was not modified.

To investigate the possible involvement of estrogenic or androgenic signals in the induction of LTP and LTD, we first analysed the induction of LTP and LTD in the presence of the block of ERs (ICI 182,780) or ARs (flutamide). Then, whether the receptor blockade affected the induction of the long-term effect we analysed the effect of inhibitors of enzyme synthesising E2 (letrozole) or DHT (finasteride).

### Data analysis and statistical evaluation

All data analysis was performed with Clampfit 10.2 (Axon Instruments, Foster City, CA, USA) and Origin 7.0 (Microcal Software, Northampton, MA, USA) software. The recordings were analyzed when the height of neuron spike was ≥ 50 mV and the resting Vm did not change more than 2-3 mV throughout the baseline. Recordings where series resistance changed by more than 20% over the duration of the experiment (30-40 min) were rejected.

Type B neurons were recognized by the shape of 1 min bins averaged AP and in particular on the presence of biphasic AHP with an ADP (Figure 1B). To characterize the drug effects on the baseline EPSP and on induction of the LTD and LTP, vestibular nerve stimulation was applied every 15 s. We measured the initial slope of EPSP using linear regression of the first 1 ms to minimize the contribution of voltage-activated conductance and used the average response recorded during a stable initial period (10 min) at the beginning of the experiments as the baseline. Modifications of the responses induced by drugs or stimulation protocols were expressed as a percentage of the baseline. Within a single experiment, we considered the mean ± SE over 2.5-min intervals before and after induction protocol application until 30 min, in order to monitor possible long-term effects. The establishment of LTP and LTD was statistically verified (Student’s paired *t* test) by comparing the values of EPSP slope at 30 min after induction protocol application with the baseline. The values of LTP and LTD amplitude given in the text are mean ± SE. The occurrence frequency of LTP or LTD (n = number of neurons) was statistically evaluated by using the chi-square (χ^2^) test. In addition, we compared the magnitude of LTP and LTD induced in different experimental conditions by the one-way analysis of variance (ANOVA). The level of significance was set at *P*<0.05 for Student’s *t* test, ANOVA, *post hoc* comparison, and χ^2^ test. Statistical analyses were performed with Statistica (StatSoft, Tulsa, OK, USA). 

## Results

### Induction of LTD and LTP in the MVN neurons

We used LIS (n = 8, 4 animals) or SIS (n = 10, 5 animals) protocols to induce LTD or LTP of the EPSP in the type B MVN neurons (Figure 1C and D).

**Figure 2:**
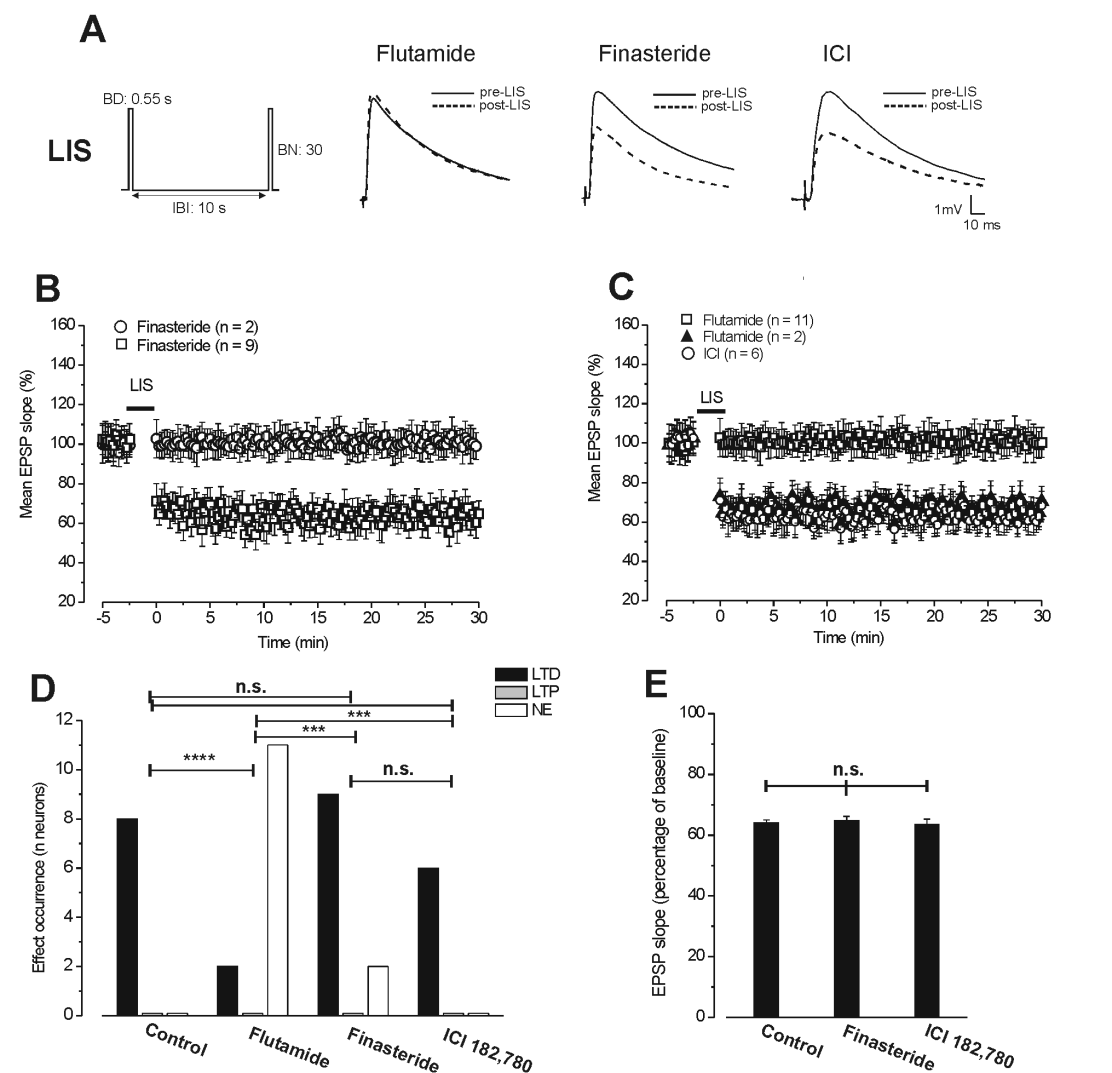
Involvement of androgens in the induction of LTD by the LIS protocol. **A**. Averaged traces (n = 20) of EPSPs evoked before (thin traces: pre-LIS) and after LIS (dashed traces: post-LIS) in the presence of flutamide, finasteride and ICI 182,780. (Left) Schematic drawing of stimulation pattern inducing LTD (LIS). **B** and **C**. Time courses of the responses induced by LIS under block of the DHT synthesizing enzyme (**B**) and of ARs or ERs (**C**). **B**. Under finasteride, LIS induces LTD (open square, n = 9) and no effect (open circle, n = 2). **C**. Under flutamide, LIS has no effect (open square, n = 11) or induces LTD (filled triangles, n = 2), while under ICI 182,780 it only induces LTD (open circles, n = 6). Note that under block of ARs (flutamide) LTD is prevented in the majority of cases, while it is still induced under block of DHT synthesising enzyme (finasteride) and of ERs (ICI 182,780). **D**. Frequency occurrence (number of neurons) of LTD (black columns), LTP (grey columns) and no effect (NE, white columns) induced by LIS in the control condition and in the presence of flutamide, finasteride and ICI 182,780 (χ^2^ test: ***P<0.005, **** *P*<0.001, n.s. = no significant). **E**. In this and in the subsequent figure columns express the mean ± SE of the EPSP slopes (percentage of baseline) within 2.5-min intervals evaluated at 30 min post-stimulus. Here, comparison between the amplitudes of LTD induced by LIS in control condition (n = 8) and in the presence of finasteride (n = 9) or ICI 182,780 (n = 6) (one-way ANOVA: n.s = no significant).

**Figure 3:**
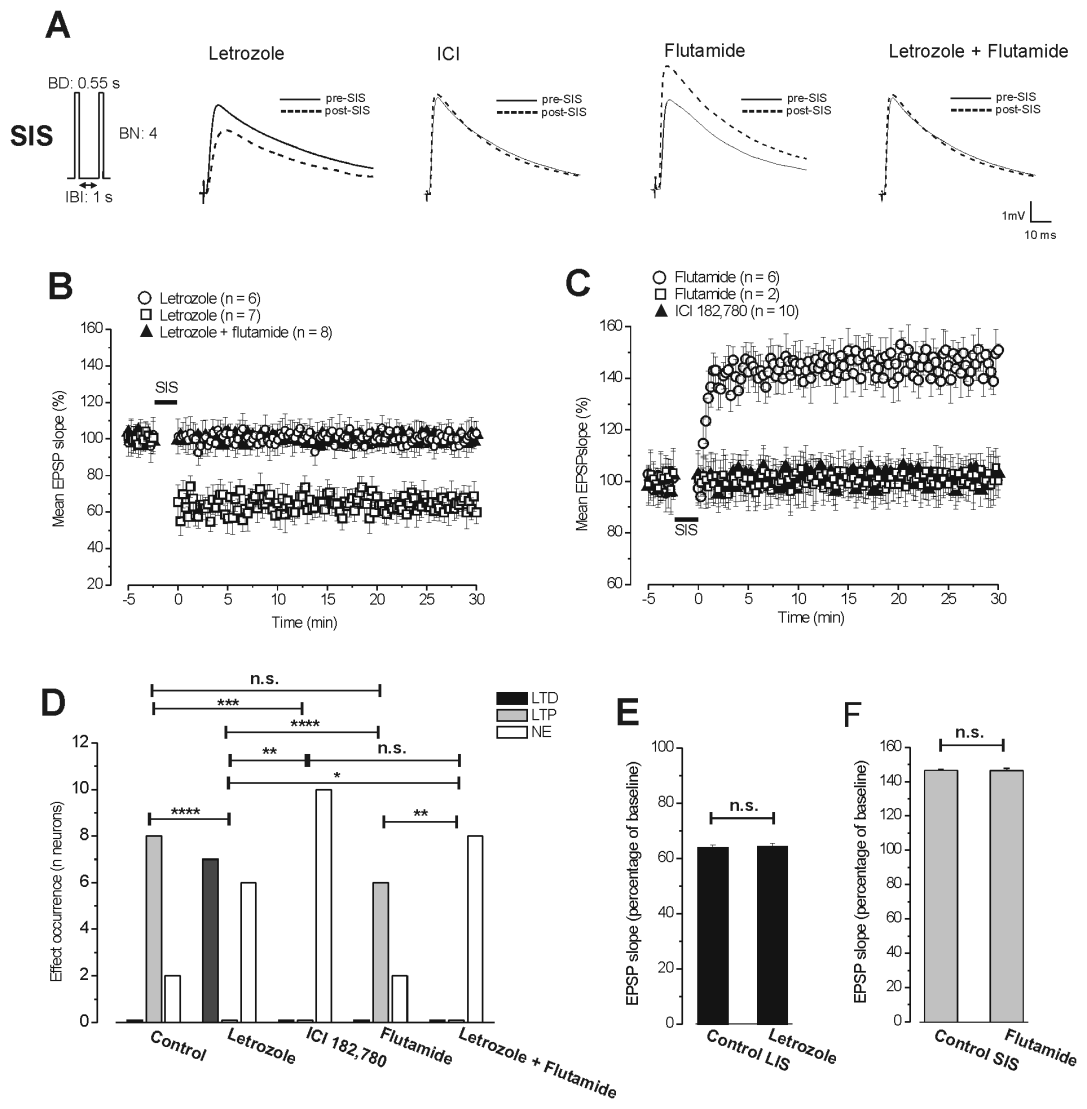
Involvement of estrogenic signal in the induction of LTP by the SIS protocol. **A**. Averaged traces (n = 20) of EPSPs evoked before (thin traces: pre-SIS) and after SIS (dashed traces: post-SIS) in the presence of letrozole, ICI 182,780, flutamide and letrozole plus flutamide. (Left) Schematic drawing of stimulation pattern inducing LTP (SIS). **B** and **C**. Time courses of the responses induced by SIS under block of the E2 synthesising enzyme (**B**) and of ERs or ARs (**C**). **B**. Under letrozole SIS induces LTD (open square, n = 7) and no effect (open circle, n = 6), while under letrozole plus flutamide it has only no effect (filled triangles, n = 8). **C**. Under ICI 182,780 SIS has no effect (filled triangles, n = 10), while under flutamide it induces LTP (open circles, n = 6) or no effect (open square, n = 2). Note that induction of LTP is fully prevented under block of ERs (ICI 182,780) while in the majority of cases it is not affected by the block of ARs (flutamide). Moreover, under inhibition of the E2 synthesising enzyme (letrozole) LTP by SIS is prevented or reverted into LTD that is abolished by flutamide. **D**. Frequency occurrence (number of neurons) of LTD (black columns), LTP (grey columns) and no effect (NE, white columns) induced by SIS in the control condition and in the presence of letrozole, ICI 182,780, flutamide and letrzole plus flutamide (χ ^2^ test: *P<0.05, **P<0.01, ***P<0.005, **** *P*<0.001 and n.s. = no significant). **E**. Comparisons between the amplitude of LTD obtained by SIS under letrozole (n = 7) and LTD normally induced by LIS in control condition (n = 8) and **F**. Comparison between the amplitudes of LTP induced by SIS in control condition (n = 8) and in the presence of flutamide (n = 6) (one-way ANOVA: n.s. = no significant).

Application of LIS protocol caused LTD reducing the EPSP slope to 64 ± 1% of the baseline in all the tested neurons (post-LIS vs pre-LIS, Student’s *t* test, *P*<0.05; Figure 1C and 2D). Conversely, SIS protocol induced LTP with an increase of the EPSP slope to 146.5 ± 0.8% in 8 neurons (post-SIS vs pre-SIS, Student’s *t* test, *P*<0.05) and no effect in the remaining 2 neurons (Figure 1D and 3D). 

The dependence of LTD and LTP on activation of the NMDAR was confirmed by applying LIS (n = 4, 2 animals) or SIS (n = 4, 2 animals) in the presence of AP-5. In all the cases, AP-5 prevented either LTD or LTP, since both stimulation protocols did not induce significant modifications of the EPSP slope compared to the baseline (LIS: 100.7 ± 0.3%, post-LIS vs pre-LIS, Student’s *t* test, *P*>0.05, SIS: 100 ± 0.7%, post-SIS vs pre-SIS, Student’s *t* test, *P*>0.05; Figure 1C and D).

### Role of sex neurosteroids in the induction of LTD and LTP

The possible involvement of AR and/or ER activation and DHT or E2 synthesis in the induction of LTD and LTP was investigated by analysing the effects of LIS and SIS protocols in the presence of the antagonists for ARs (flutamide) or ERs (ICI 182,780) and of inhibitors of 5α-reductase (finasteride) or P450-aromatase (letrozole).

### Androgens but not E2 are involved in the induction of LTD

#### Effect of the block of androgenic pathway on the induction of LTD

To verify whether LTD induced by the LIS protocol could depend on the androgenic neurosteroids we first delivered this stimulation pattern under the block of the ARs (flutamide: n = 13, 5 animals). Application of flutamide did not modify the baseline EPSP (101.2 ± 0.56%, Student’s *t* test, *P*>0.05) but in the presence of flutamide no effect was observed in 11 neurons (101.1 ± 0.75%, post-LIS vs pre-LIS, Student’s *t* test, *P*>0.05) while LTD (65.7 ± 2.2%, post-LIS vs pre-LIS, Student’s *t* test, *P*<0.05) was still induced in only 2 cases (Figure 2A, C, D and E). In order to verify the possible involvement of local synthesis of DHT in the induction of LTD we analysed the effect of LIS under the blockade of 5α-reductase with finasteride (n = 11, 5 animals). Finasteride had no effect on the baseline EPSP (99.8 ± 0.36%, Student’s *t* test, *P*>0.05) but, unlike flutamide, it did not affect LTD in most of the cases. In fact LTD (64.8 ± 1.4%, post-LIS vs pre-LIS, Student’s *t* test, *P*<0.05) was induced in 9 neurons, while no effect was observed in the other 2 neurons (Figure 2A, B, D and E). As a result of this different influence of the drugs, the occurrence frequency of LTD under flutamide was significantly different compared with finasteride and control, while the occurrence of LTD under finasteride did not differ from the control (flutamide vs control: χ^2^ = 13.9, df = 2, *P*<0.001; finasteride vs control: χ^2^ = 1.37, df = 2, *P*>0.2; flutamide vs finasteride: χ^2^ = 10.5, df = 2, *P*<0.005; Figure 2D). In addition, the amplitude of LTD under finasteride was not statistically different from the LTD observed in control condition and LTD induced by LIS under ICI 182,780 (see below) (ANOVA: F_1,20_ = 0.1, *P*=0.8; Figure 2B, C and E).

#### Effect of the block of estrogenic pathway on the induction of LTD

We also assayed the possible influence of estrogenic signal activation on the LIS dependent LTD by blocking the ERs with ICI 182,780 (n = 6, 3 animals). ICI 182,780 did not modify the baseline EPSP (100.5 ± 0.55%, Student’s *t* test, *P*>0.05) and had no effect on the induction of LTD. In fact, in the presence of ICI 182,780 LTD (63.6 ± 1.7%, post-LIS vs pre-LIS, Student’s *t* test, *P*<0.05) was obtained in all the examined neurons (Figure 2A, C, D and E). The occurrence of LTD was not different from that observed under control condition and in the presence of finasteride, but it was different from that obtained under flutamide (ICI vs control: χ^2^ = 0.008, df = 2, *P*=1; ICI vs finasteride: χ^2^ = 1, df = 2, *P*>0.2, ICI vs flutamide: χ^2^ = 11.7, df = 2, *P*<0.005; Figure 2D). As stated above, the amplitude of LTD obtained under ICI 182,780 was not statistically different from that induced under finasteride and in control condition (ANOVA: F_1,20_ = 0.1, *P*=0.8; Figure 2B, C and E).

### E2, but not androgens, is involved in the induction of LTP

#### Effect of the block of estrogenic pathway on the induction of LTP

Our previous study demonstrated by extracellular field potential recordings that the local synthesis of E2 and activation of ERs play a key role in the induction of LTP by HFS in the MVN neurons [28]. Thus, we verified this result by analysing the effect of ICI 182,780 (n = 10, 4 animals) and letrozole (n = 13, 5 animals) on the induction of LTP by the SIS protocol in single MVN neurons. Like observed for ICI 182,780 (see above), the baseline EPSP was not affected by letrozole application (100.3 ± 0.72%, Student’s *t* test, *P*>0.05). However, the effects induced by the SIS protocol were completely different in the presence of ICI 182,780 or letrozole. In fact, while ICI 182,780 prevented LTP in all the examined neurons (100.1 ± 0.78%, post-SIS vs pre-SIS, Student’s *t* test, *P*>0.05), letrozole abolished LTP in only 6 neurons (100.6 ± 0.84%, post-SIS vs pre-SIS, Student’s *t* test, *P*>0.05) while in the other 7 neurons SIS induced LTD (64.4 ± 1.1%, post-SIS vs pre-SIS Student’s *t* test, *P*<0.05; Figure 3A, B, C, D and E). Because of this difference in the drug effects, the occurrence frequency of the SIS effects under ICI 182,780 or letrozole was significantly different from that observed in control condition and between them (ICI vs control: χ^2^ = 13, df = 2, *P*<0.005; letrozole vs control: χ^2^ = 16.2, df = 2, *P*<0.001; letrozole vs ICI: χ^2^ = 7.4, df = 2, *P*<0.01; Figure 3D). Concerning the amplitude of LTD induced by SIS in the presence of letrozole it was not significantly different from that normally induced by the LIS protocol in control condition (see above, ANOVA: F_1,13_ = 0.068, *P*=0.79; Figure 3E).

#### Effect of the block of androgenic pathway on the induction of LTP

The possible effect of androgenic signal activation on the induction of LTP was analysed by applying SIS protocol under the blockade of ARs with flutamide (n = 8, 4 animals). Under flutamide SIS induced LTP (146.3 ± 1.3%; post-SIS vs pre-SIS, Student’s *t* test, *P*<0.05) in 6 neurons and had no effect (100.8 ± 0.98%, post-SIS vs pre-SIS, Student’s *t* test, *P*>0.05) in the remaining 2 neurons. The occurrence and amplitude of LTP under flutamide did not significantly differ from those observed under control condition (χ^2^ = 0.06, df = 2, *P*>0.95; ANOVA: F_1,12_ = 0.1; *P*=0.75; Figure 3A, C, D and F). We also verified whether LTD resulting by SIS under letrozole depended on androgenic signals by applying SIS protocol in the presence of combined blockade of P450-aromatase and ARs (letrozole plus flutamide, n = 8, 3 animals). The combined block prevented either LTP or LTD (100.5 ± 0.56%, post-SIS vs pre-SIS, Student’s *t* test, *P*>0.05; Figure 3A, B and D) so that the occurrence of SIS effects in this condition (only no effects) was statistically different from that obtained under letrozole alone (LTD and no effects) or flutamide alone (LTP and no effects) and similar to that obtained under ICI 182,780 (only no effects) (letrozole plus flutamide vs letrozole: χ^2^ = 6.1, df = 2, *P*<0.05; letrozole plus flutamide vs flutamide χ^2^ = 9.3, df = 2, *P*<0.01; letrozole plus flutamide vs ICI: χ^2^ = 0.004, df = 2, *P*=1; Figure 3D).

## Discussion

In this study we demonstrate for the first time in the vestibular system that the long-term synaptic changes induced in the MVN neurons of male rat by afferent stimulations and their direction depend on different sex neurosteroid signals, since the LTD requires the presence of androgens, T or DHT, and LTP that of the estrogen E2. In fact, the blockade of ARs with flutamide prevented in almost all cases the induction of LTD by LIS, while that of ERs with ICI 182,780 impeded LTP elicited by SIS. Conversely, LTD and LTP were not modified by ICI 182,780 and flutamide, respectively.

These effects under blockade of the sex steroid receptors imply the local synthesis of androgens and estrogens. Indeed, under letrozole that prevents the conversion of T into E2 by blocking the P450-aromatase [7,10-14] the induction of LTP by SIS was impeded and LTD instead of LTP was often observed. Conversely, finasteride that blocks the synthesis of DHT from T, through inhibition of the 5α-reductase enzyme [8,9,13], did not abolish LTD in response to its inducing stimulation protocol (LIS), except in two cases. In general, these results confirm the crucial role of the local synthesis of E2 in the induction of LTP previously observed in the MVN neurons [28], but do not provide a definitive demonstration for the involvement of the DHT synthesis in the induction of LTD. 

Concerning the E2, the P450-aromatase may play a role either by synthesizing E2 in response to stimulation inducing LTP or by synthesizing E2 continuously to permit to a specific stimulus for LTP to be effective. However, the permissive action of E2 is doubtful since the same specific stimulation for LTP can lead to LTD under the P450-aromatase block. This supports the idea that the afferent stimulation is not specific *per se*, but for its capability to activate the synthesis of E2. Since we know that LTP can be induced in the MVN by simply increasing the level of E2, through exogenous administration [24], it is likely that the stimulation activates the synthesis of E2, and this, in turn, leads to LTP. In this view, the inversion of LTP into LTD that occurs in the presence of letrozole, but not of ICI 182,780, can be explained by a probable accumulation of upstream and/or downstream androgenic metabolites that takes place when the transformation of T into E2 is impeded. Therefore, LTD might result from activation of ARs by the increased level of androgens or by an interaction of DHT metabolites, like androstane-dioles, with other receptors as GABA_A_ or ERβ [42,43]. However, the finding that flutamide fully prevented the LTD observed under block of the estrogenic pathway strongly suggests the involvement of AR activation by T and/or DHT. In addition, an effect on GABA_A_ receptor can be certainly ruled out since we worked under block of GABAergic transmission. The possible influence of androstane-dioles on ERβ could also raise the suspicion of a role of this pathway in the induction of LTP by SIS, but activation of ERs by locally synthesized E2 appears to be the only responsible for LTP since under letrozole LTP was abolished or inverted into LTD. 

Moreover, the evidence that E2-dependent LTP or androgen-dependent LTD can be obtained in the same MVN neurons also suggests that they have the possibility to develop either potentiation through E2 or depression through T or DHT depending on the afferent stimulation pattern. Therefore, the mechanisms for inducing LTP and LTD by sex neurosteroids seem to coexist in many MVN neurons, like supported by our recent immunohistochemical evidence of co-localization of ERs and ARs in the majority of them [25]. 

Concerning the involvement of the DHT synthesis in LTD, our findings do not yet lead to its demonstration, since under inhibition of the 5α-reductase LTD was still induced, even though it was prevented by the AR blockade in almost all cases. This suggests that, although the activation of androgen signaling is required for LTD, the conversion of T into DHT could not be necessary. However, we know that 1) DHT is able to induce LTD in the MVN [23,25], 2) under block of the DHT synthesis the occurrence of LTD by exogenous administration of T was reduced [23] and 3) DHT shows a greater affinity for ARs than T [41,44]. Taken together these results put forward the conversion of T into DHT during the LTD-inducing stimulation. In this view the maintenance of LTD under the block of 5α-reductase can be explained by the upstream accumulation of T substituting for the lack of DHT. However, according with our previous field potential study with exogenous administration of T [23], we expected that accumulating T not only reduced the probability of LTD, but also increased that of LTP, owing to its transformation into E2. But this did not occur in the present experimental condition. It may be that these different effects depend on a lower level of T elicited by the synaptic stimulation compared to that reached through exogenous administration of T [23], or to an inhibitory influence of the LIS pattern on the P450-aromatase. Therefore, we hypothesize that LTP can only be induced when the synthesis of E2 is activated by a specific pattern of stimulation. On the contrary, in theory LTD could be elicited by any stimulation able to increase the level of T, but not to activate the P450-aromatase. Therefore, the neural synthesis of E2 seems to be crucial for guiding vestibular synapses toward LTP or LTD. In fact, we suggest that both inducing LTP and LTD stimulation facilitates the synthesis of T, but only the specific activation of P450-aromatase can drive the direction of synaptic change toward the potentiation. It remains to be demonstrated whether the transformation of T into DHT is also guided by specific stimulation patterns. 

The mechanism by which stimulation patterns may lead to production of different neurosteroids is at the moment fully unknown. We know that the E2 synthesis may be mediated by the Ca^2+^ influx through the NMDAR [11] and activation of the P450-aromatase [12-14,26,27]. Similarly, NMDAR dependent Ca^2+^ influx elevates the level of pregnenolone and pregnenolone sulphate [11], the first precursors of sex neurosterogenesis [5,6]. But, no evidence for a Ca^2+^-driven activation of 5α-reductase has been provided so far. Our electrophysiological findings may only prompt for a role of different levels of Ca^2+^ associated with different synaptic stimulations in the specific activation of estrogenic or androgenic enzymatic conversion of T. Moreover, since both LTP and LTD in the MVN are NMDAR dependent phenomena, and induction of LTP or LTD is mediated by different amount of NMDAR-dependent Ca^2+^ signalling [45], we suggest that these different levels of Ca^2+^ allow the synthesis of potentiating or depressant neurosteroids, that in turn regulate the NMDAR function [24,27,46-50]. However, the Ca^2+^ dynamics associated with stimulation patterns inducing LTD or LTP in the vestibular nuclei need to be explored in detail, as well as the possible effect of different Ca^2+^ levels in activating the estrogenic or androgenic signals involved in the NMDAR dependent synaptic plasticity. The intracellular signalling pathways from ERs and ARs to NMDAR that are implied in the rapid synaptic effects of E2 and T-DHT have not been well elucidated, but activation of different kinase signal cascades including mitogen-activated protein kinase pathway has been proposed [3,27,40,47,50].

On the whole, this study suggests that the activity dependent neural production of E2 and T-DHT plays a very important role in determining the sign of vestibular synaptic plasticity. However, the sex neurosteroids might influence this plasticity in a different way considering that their effects, as well as the activity of their synthesising enzymes, may be affected by the level of circulating hormones depending on sex, estrous cycle and age [33,34]. In fact, significant morphological and functional modifications of neuronal circuitry have been reported to depend on the history of estrogen and androgen impact on the neurons [51]. A further complexity in figuring out the real effects of sex neurosteroids in a more integrated system comes from their possible role in modulating inhibitory GABAergic and glycinergic inputs. However, even though our previous studies with exogenous administration of E2 and DHT show no substantial changes in their effects caused by GABA [23,28], we actually found that induction of LTP in the MVN is facilitated by the E2 mediated reduction of GABAergic transmission [28]. Concerning LTD, it is likely that in the presence of GABAergic transmission it may be facilitated by the downstream DHT metabolites acting on GABA_A_ receptors [42]. 

Nevertheless, even if performed in a simplified experimental condition our study gives clear evidence that the sign of glutamate synaptic plasticity can be determined by specific stimulation patterns in dependence on the local activation of estrogenic or androgen pathways 

## Conclusions

On the whole this study demonstrates a clear role of sex neurosteroids in the induction of vestibular synaptic plasticity. In particular, it demonstrates a distinct role of E2 through ERs, and T or DHT through ARs, in mediating the induction of LTP and LTD, respectively. Therefore, we suggest that the synaptic stimulation pattern is responsible for the induction of LTP or LTD since it drives the synthesis, at a neural level of estrogens or androgens. In this context, neural E2 and T-DHT seem to be very effective modulators of synaptic plasticity that can significantly contribute to vestibular learning processes.
